# Lessons learned from a home-based exercise program for adolescents with pre-existing chronic diseases during the COVID-19 quarantine in Brazil

**DOI:** 10.6061/clinics/2021/e2655

**Published:** 2021-06-23

**Authors:** Isabela Gouveia Marques, Camilla Astley, Sofia Mendes Sieczkowska, Amanda Yuri Iraha, Tathiane Christine Franco, Fabiana Infante Smaira, Bruna Caruso Mazzolani, Luana Cristina do Amaral Miranda, Bianca Pires Ihara, Rosa Maria R. Pereira, Ligia Bruni Queiroz, Clovis Artur Silva, Bruno Gualano

**Affiliations:** I Grupo de Pesquisa em Fisiologia Aplicada e Nutricao, Escola de Educacao Fisica e Esporte, Universidade de Sao Paulo, Sao Paulo, SP, BR; II Divisao de Reumatologia, Hospital das Clinicas HCFMUSP, Faculdade de Medicina, Universidade de Sao Paulo, Sao Paulo, SP, BR; III Instituto da Crianca e do Adolescente (ICr), Hospital das Clinicas HCFMUSP, Faculdade de Medicina, Universidade de Sao Paulo, Sao Paulo, SP, BR; IV Centro de Pesquisa em Alimentos, Universidade de Sao Paulo, Sao Paulo, SP, BR

Brazil has been among the top three countries most affected by the novel coronavirus disease (COVID-19), with more than 14 million people infected and 410,000 deaths as of early May 2021 (1,2). Quarantine and social distancing measures have been recommended to reduce the spread of COVID-19 in Brazil since March 2020 (1-3). It was estimated that school closures affected more than 52 million Brazilian children and adolescents (4). The COVID-19 pandemic has impacted the lifestyle of citizens due to measures such as school closures, extended home confinement, and limited access to physical activities (5-11). These changes also extend to the pediatric population. A survey of 495 Brazilian children and adolescents revealed that school closures and home confinement, due to the COVID-19 pandemic, were responsible for a decrease in daily physical activity. Approximately one-third of respondents met the physical activity guidelines before the pandemic, but only two out of ten met the guidelines during the quarantine (12).

These lifestyle behavior changes could have long-term impacts on the physical and mental health of children and adolescents (5,6,11,13,14). This scenario is worse for adolescents with pre-existing chronic diseases as they are at an increased risk of infection and their regular care management is temporarily suspended (5,15-17).

## Research agenda

Researchers at the Children and Adolescent’s Institute of our large university and tertiary hospital designed a multidisciplinary and multi-professional study to investigate the impact of the COVID-19 quarantine on the lifestyle habits of adolescents with pre-existing chronic diseases, including gastrointestinal and liver, rheumatological, nephrological, endocrinological conditions, and those with liver and kidney transplantation. Our team developed and delivered a 12-week home-based exercise program to mitigate the deleterious effects of reduced physical activity due to the quarantine, based on the fact that exercise can improve both physical and psychosocial well-being, as well as health-related quality of life parameters (18,19).

### Home-based exercise program

Based on state-of-the-art recommendations (20-22), the home-based exercise program included aerobic and bodyweight exercises, such as jumping jacks, squats, push-ups, sit-ups, planks, and dynamic planks. This program was offered in two intensities - beginner and intermediate - according to the patient’s previous experience with the exercise, and its complexity. In addition, studies have shown that digital platforms can increase adolescents’ adherence to aerobic and strengthening exercises during lockdown by four times compared to non-users (23).

Active outpatients were invited to participate in the study by the attending physician. Physical activity sessions were not included in their regular care management. Adolescents aged 10 to 18 years with inflammatory, autoimmune, or metabolic diseases or chronic diseases such as gastrointestinal and liver, rheumatological, nephrological, or endocrinological conditions, as well as those with kidney and liver pediatric transplantation, all of which require multiple immunosuppressive and biological drugs, were evaluated.

Patients received instructional videos, photos, and GIFs demonstrating and describing the exercises. A video call was conducted prior to the program initiation to provide details about the program and to collect information on the patient’s health status. Online supervised group sessions were scheduled every two weeks. Patients were required to complete the training sessions three times a week and report their adherence using Redcap^®^. A total of 220 patients were enrolled in the study. This study was registered at ClinicalTrials.gov (protocol record 31314220.5.0000.0068) and was approved by the Brazilian National Committee for Research Ethics (CONEP, number 4.081.961).

## Lessons learned

The home-based exercise program was offered as a health promotion and coping strategy to overcome the negative effects of the COVID-19 pandemic on the physical and mental health of adolescents with chronic disease. We describe the challenges faced during program delivery and the strategies utilized by our team. We aim to inform and educate practitioners, interventionists, and clinicians, planning to deliver home-based exercise programs for adolescents, while in-person exercise sessions are not recommended because of public health safety measures.

1. Increased online activity
Challenge: As quarantine orders were implemented, most activities had to be adjusted to the online format. Some patients were overwhelmed by online activities such as mandatory educational sessions, telemedicine appointments, and social relationship interactions via digital media, and perceived this program as another online obligation instead of as an opportunity to improve health (24-26). Therefore, many patients reported losing interest in the program after the initial weeks. The patients reported being stressed by staying at home. Digital saturation, boredom, and loneliness are possible explanations for the loss of interest in the program (27).
Innovation: The home-based exercise program requires online interactions. The online supervised group sessions were held once every other week, to reduce the burden on participants. However, participants were expected to report adherence three times per week using REDCap^®^. The most important action taken to reduce the online burden was modifying the platform used to deliver the program. Initially, we used Google Meet for the supervised online sessions and the online research tool REDCap^®^ to track adherence. Our team quickly identified that learning to navigate two new tools was difficult for the participants and hence, we started using WhatsApp^®^, a popular communication software application in Brazil, to communicate with the participants and to deliver the exercise program. WhatsApp^®^ allows users to exchange text and audio messages, photos, and videos and to make phone and video calls, along with many other features. In addition to communication with the research team, patients used WhatsApp^®^ as a program delivery tool via video calls and to improve program adherence. Two patients chose not to share their videos during the supervised online sessions; therefore, they reported and sent videos while exercising as proof of adherence through personal messages. Other patients recorded and sent videos while exercising to inquire about their posture during exercise. WhatsApp^®^ allowed for flexibility in the timing and form of communication with the research team to report adherence (message, audio, photo, or video). Patients might not have been able to use REDCap^®^ for this purpose.
2. Multiple scenarios during quarantine
Challenge: The group was heterogeneous, including early- and late-onset adolescents with various chronic conditions. Each patient and their family faced the quarantine uniquely. In pre-pandemic times, it was known that “one size does not fit all” when it comes to physical activity engagement, and that also holds true during quarantine. For example, some patients rigorously followed stay-at-home orders, while others had to work to contribute to their family’s income and, therefore, had limited time to participate in the home-based exercise program. Some patients received full support from family members to continue engaging in educational and health-related activities, while others faced hostile situations in the home environment.
Innovation: Our team implemented a video call system to determine the overall context of a patient’s home life. During this call we, 1) introduced the researcher responsible for delivering the exercise training program, 2) inquired about physical activity and health status, 3) provided detailed instructions for the exercises, and 4) observed and provided feedback on the patient’s movements and assisted with body awareness. We informally and gently enquired about the home environment, the stresses faced by the patient and, the different coping mechanisms used by the adolescent to deal with the quarantine and stress. We explained the benefits of the exercise program on the physical and mental health of the patient. We aimed to provide an individualized and tailored program to meet each patient’s needs. Our team also served as a link to the medical team forwarding inquiries about appointments, medications, symptoms, and psychological well-being.
3. Lack of social support
Challenge: Social support is an important predictor of engagement in physical activity. Some patients, especially those who were younger, or had no experience with formal exercise training or the benefits of regular exercise, required more support than others. Patients who received social support from their parents were more likely to successfully complete the exercise program. Patients with family members working full-time demonstrated a greater need for accountability. One patient requested immediate assistance due to an extremely stressful home environment and was referred to psychological care and follow-up.
Innovation: We tried to identify an immediate family member who could provide social support for the patients. Some parents and siblings provided successful support and encouraged participation and at times, agreed to exercise with the patient. The research assistant provided closer attention to patients who had no immediate support. We offered additional online supervised sessions on patients’ requests. One patient requested that all three weekly sessions be supervised due to her lack of motivation, previous experience with formal exercise, and the need for accountability. Patients appreciated the individualized attention from a qualified exercise professional (22).
4. Internet access and connectivity, and smartphone ownership
Challenge: Some patients had no access to the digital tools required for the online exercise program. Younger adolescents usually relied on their smartphones to connect to the online sessions. Poor Internet connection also limited the participation of some patients.
Innovation: Our team aimed to provide an individualized experience. The frequency of the supervised online sessions was modified, and sessions were rescheduled according to the patient’s needs. If the patient relied on a parent’s smartphone, we either arranged with the parent for a suitable day and time or arranged for the sessions to be accessed through a computer. Unfortunately, dropout was likely when the patient depended on someone else. In cases where the internet connection would not allow the patient to complete a session, they were asked to record a video, so that we could provide proper feedback on posture.

## Recommendations

We recommend that practitioners, interventionists, and clinicians planning to deliver home-based exercise programs for adolescents should make the program as individualized as possible. We do understand staff and budget limitations. However, the quarantine scenario that adolescents face is unique. With multiple stressors playing a role in their physical and mental health, we suggest that every effort be made to tailor the program to adolescents’ needs. We recommend the following based on our observations in this study:

1. Try to understand the overall context involving the adolescent with a chronic condition.
2. Let the adolescent suggest the frequency and amount of monitoring needed.
3. Check the adolescent’s access to digital tools.
4. Learn about physical limitations, pre-pandemic levels of physical activity and exercise preferences.
5. Adapt and tailor the exercises.
6. Aim to increase motivation without pushing too much.

Due to the urgent scientific call for home-based exercise programs to be delivered during the quarantine of the COVID-19 pandemic (11), our study methods did not use a participatory design and, therefore, researchers were unable to include potential participants and other stakeholders in the intervention design. Although some patients discontinued the exercise program, many achieved the benefits of regular exercise.

In conclusion, we believe that the pandemic has dramatically changed lifestyle behaviors for most people, especially quarantined adolescents with pre-existing chronic conditions. We also believe that home-based delivery can reach vulnerable people, which is important, especially considering the asymmetric impact of the pandemic on socioeconomically deprived and unhealthy groups (28). Interventionists could follow the recommendations presented in this manuscript, to meet the patient’s needs during quarantine. By understanding adolescents’ context and allowing them to guide the various factors involved in intervention delivery, chances of success are increased.

## AUTHOR CONTRIBUTIONS

All authors contributed substantially to the conception and design of the study, and in the analysis and interpretation of data. All authors critically revised and approved the final version of the manuscript.
